# Impact of maternal, infant, and household factors on early-life gut microbiome development in a rural setting

**DOI:** 10.1093/ismejo/wrag124

**Published:** 2026-05-13

**Authors:** Mona Parizadeh, Isabelle Laforest-Lapointe, Angélica Serrano-Vázquez, Patricia Morán-Silva, Liliana Rojas-Velázquez, Javier Torres, Cecilia Ximénez-García, Marie-Claire Arrieta

**Affiliations:** Department of Physiology & Pharmacology and Pediatrics, Cumming School of Medicine, University of Calgary, Calgary, AB T2N 4N1, Canada; International Microbiome Centre, University of Calgary, Calgary, AB T2N 4N1, Canada; Snyder Institute for Chronic Diseases, University of Calgary, Calgary, AB T2N 4N1, Canada; Alberta Children’s Hospital Research Institute, University of Calgary, Calgary, AB T2N 4N1, Canada; Département de Biologie, Université de Sherbrooke, Sherbrooke, QC J1K 2R1, Canada; Centre de recherche du CHUS, Sherbrooke, QC J1H 5N4, Canada; Laboratorio de Inmunología del Departamento de Medicina Experimental, UNAM, Mexico City, 06720, Mexico; Laboratorio de Inmunología del Departamento de Medicina Experimental, UNAM, Mexico City, 06720, Mexico; Laboratorio de Inmunología del Departamento de Medicina Experimental, UNAM, Mexico City, 06720, Mexico; Unidad de Investigación en Enfermedades Infecciosas, UMAE Pediatría, IMSS, Mexico City, 06720, Mexico; Laboratorio de Inmunología del Departamento de Medicina Experimental, UNAM, Mexico City, 06720, Mexico; Department of Physiology & Pharmacology and Pediatrics, Cumming School of Medicine, University of Calgary, Calgary, AB T2N 4N1, Canada; International Microbiome Centre, University of Calgary, Calgary, AB T2N 4N1, Canada; Snyder Institute for Chronic Diseases, University of Calgary, Calgary, AB T2N 4N1, Canada; Alberta Children’s Hospital Research Institute, University of Calgary, Calgary, AB T2N 4N1, Canada

**Keywords:** early-life microbiome, microbial succession, microbial diversity, inter-kingdom co-occurrences, Featured image

## Abstract

Early-life gut microbiome development is influenced by host, microbial, environmental, and social factors. Rural infants typically exhibit greater microbial diversity than their urban counterparts, yet microbiome maturation patterns in less industrialized settings remain underexplored. Additionally, though microbial eukaryotes are integral to gut ecology, most studies to date have focused predominantly on bacterial communities. Using shallow shotgun metagenomics and 18S ribosomal RNA gene sequencing, we characterized bacterial and eukaryotic gut microbiomes in an intensively sampled longitudinal cohort of 10 infants from a rural community in Morelos, Mexico, each followed monthly from the first to the 18th month, providing a detailed view of early-life microbiome development in a low-resource setting. Although both bacterial and eukaryotic alpha diversity increased over time, they showed distinct colonization trajectories. Age, delivery mode, and environmental exposures, such as animal contact and household factors, influenced bacterial and eukaryotic community compositions, and bacterial metabolic composition. Inter-kingdom microbial networks varied with age, with a reduction in taxonomic diversity after the first year of life. Age and birth mode also influenced changes in the overall community structure and connectivity of microbial co-occurrence patterns, but did not impact the associations among specific microbial taxa. Functional profiling revealed that bacterial metabolic potential diversified with age, whereas the mode of birth had a minimal impact on functional variation. These findings highlight the dynamic nature of bacterial and eukaryotic microbiota in early life and underscore the need to explore how rural environmental exposures shape microbial maturation, with potential implications for immune development and long-term health.

## Introduction

Microorganisms start colonizing the human gut during birth and undergo substantial changes during the first 2–3 years of life, a critical window for gut microbiome development [1, 2]. The early-life gut microbiome is shaped by interactions between host factors, maternal influences, and environmental exposures. Understanding these processes is essential for elucidating the foundations of human health and disease.

Social geography—the influence of social environments on human life—and maternal factors play a significant role in shaping the early-life gut microbiome. Infants in rural settings exhibit higher microbial species richness compared to their urban counterparts, likely due to greater microbial exposure in less industrialized environments [1, 3]. These differences in microbial colonization are influenced by varied environmental factors, including greater frequency of animal contact, dietary patterns, and limited access to medications [4, 5]. Maternal factors, such as mode of birth, also contribute to initial microbial colonization [1, 6]. Vaginally delivered infants acquire microbes from the maternal vaginal canal, skin, and gut, leading to distinct colonization patterns compared to cesarean-section (C-section) delivered infants, who primarily acquire microbes from maternal skin and the hospital environment [7, 8]. Vaginal delivery is associated with higher microbial diversity and the enrichment of beneficial taxa such as *Bifidobacterium* and *Bacteroides*, whereas C-section delivery is linked to reduced diversity and delayed microbial colonization, which may increase susceptibility to immune and metabolic disorders in early life [9]. These represent short-term differences, as many delivery-mode–associated microbial signatures diminish after the introduction of complementary foods [10]. These early differences, alongside environmental factors such as animal exposure, dietary transitions, and rural vs urban living, provide a framework for understanding the drivers of gut microbiome diversity during early life.

Even though most studies have focused on bacterial communities, non-bacterial microbial populations such as fungi and protists also play critical roles in gut ecosystem function and host–microbe interactions [8–11]. Microbial eukaryotes, including fungi like *Candida, Malassezia*, and *Aspergillus*, colonize the gut shortly after birth and interact with bacterial communities to shape microbiota composition and function [12, 13]. These inter-kingdom interactions, although poorly understood, may have important implications for early-life health and development [12]. For example, previous studies have linked the presence of *Blastocystis* sp. in the human gut with increased bacterial alpha diversity [14] and compositional changes of abundant bacterial taxa [15, 16]. However, the role of eukaryotic microbes in rural populations and their co-occurrence patterns with bacteria remain underexplored.

To address these gaps, the present study examines the gut microbiome of a unique longitudinal cohort of 10 infants from a rural area in Morelos, Mexico, sampled monthly from the first to the 18th month of age. Using metagenomic and 18S ribosomal RNA (rRNA) gene sequencing, we characterize the taxonomic and functional profiles of the bacterial and eukaryotic microbiomes, assess their temporal dynamics, and evaluate the influence of mode of delivery and environmental factors on their composition. This study contributes to understanding the interplay between bacterial and eukaryotic communities in early-life microbiome development, highlighting the significance of rural environments in shaping gut microbial ecosystems. By integrating bacterial and eukaryotic datasets, we provide insights into inter-kingdom microbial co-occurrence patterns and their role in gut health during early life.

## Materials and methods

### Study design and sample collection

We conducted a longitudinal human cohort study in a rural district in Xoxocotla, in the state of Morelos, central Mexico. Xoxocotla, is officially classified as an indigenous community with a predominantly rural character. The municipality has a population of ~20 000 residents, with many households engaged in small-scale agriculture, informal labor, and local commerce. Access to piped water and sanitation is variable, and most families live in multigenerational households. Dietary practices include traditional maize-based foods, and breastfeeding rates remain high relative to urban Mexican populations. These characteristics distinguish the community from large metropolitan centers and reflect a rural, lower-income context.

Stool samples were collected from 10 healthy infants who had no chronic disease or acute infection at the time of enrollment. These infants were born between March 2015 and May 2016. Infant samples were collected monthly from the first to 18th month of age, with the first sampling occurring during the second to fourth weeks of birth. Variables collected for each infant included mode of birth, birth date, sex, age at the time of sampling (in months), weight, height at various sampling times, weaning age (defined as age when breastfeeding stopped), presence of parasites, vaccination history, and breastfeeding status. All infants were breastfed. The distribution of all variables in this cohort, including breastfeeding status, across infants reflects the characteristics of the recruited cohort and was not predetermined. We categorized the samples into three age groups: 1–6 months, 7–12 months, and 13–18 months. These intervals were selected to reflect key developmental stages during infancy: exclusive breastfeeding (1–6 months), the introduction and expansion of complementary feeding (7–12 months), and the transition toward a more adult-like diet and microbiome maturation (13–18 months). Additional data were gathered on maternal education and occupation, housing conditions (e.g. construction material, number of rooms, and household size), access to water and its type, energy sources, and exposure to different types of animals (including pets, barn animals, rodents, and cockroaches). Samples were stored at −80°C until required and subsequently shipped to Microbiome Insights in Vancouver, Canada, for DNA extraction, library preparation, and sequencing.

#### Ethics approval

Research Ethics approvals were obtained from Universidad Autónoma de México (FM/DI/014/2019) and the University of Calgary (REB18-1271) to conduct this study. Participant families were invited to the study, and only those who provided written consent were included.

### DNA extraction

At Microbiome Insights, DNA extraction was carried out on ~0.1 g of stool samples on a KingFisher robot (Thermo Fisher Scientific, Waltham, MA, USA) using the MagAttract PowerSoil DNA KF kit (Qiagen, Hilden, Germany). The extraction was performed according to the manufacturer’s instructions. Prior to storing extracted DNA samples at −20°C, DNA quality was evaluated visually via gel electrophoresis and quantified using a Qubit 3.0 fluorometer (Thermo Fisher Scientific, Waltham, MA, USA).

### Quantitative polymerase chain reactions

To quantify the total microbial content in each sample, we performed quantitative polymerase chain reactions (qPCR) on genomic DNA isolated from stool samples using bacterial 16S and eukaryotic 18S rRNA gene-specific primers and the QIAamp Fast DNA Stool Mini Kit (Qiagen, Hilden, Germany). All qPCRs were conducted in triplicate for stool samples that contained sufficient DNA, using the 7500 Fast Real-Time System (Applied Biosystems, Life Technologies, Carlsbad, CA, USA). The template DNA used in the qPCR was derived from stocks normalized to 1 ng/μl and diluted as needed. For 16S rRNA gene quantification, each 10 μl reaction contained 5 μl of iQ SYBR Green Supermix, 0.5 μl of each of the universal 16S-FR (5*′-TCC TAC GGG AGG CAG CAG T-*3*′*) and 16S-RV (5*′-GGA CTA CCA GGGTAT CTA ATC CTG TT-*3*′*) [17] primers, 2 μl of nuclease-free water, and 2 μl of 1 ng/μl genomic DNA. The qPCR program included an initial step of 5 min at 94°C, followed by 40 cycles of 15 s at 94°C, 30 s at 60°C, and 30 s at 72°C, with a final cycle of 15 s at 95°C, 60 s at 60°C, 15 s at 95°C, and 15 s at 60°C. For 18S rRNA gene quantification, each 20 μl reaction contained 10 μl of iQ SYBR Green Supermix, 2.5 μl of each of the FR1 (5*′-AIC CAT TCA ATC GGT AIT-*3*′*) and FF390 (5*′-CGA TAA CGA ACG AGA CCT-*3*′*) [18] primers, 3 μl of nuclease-free water, and 2 μl of 1 ng/μl genomic DNA. The qPCR program consisted of an initial step of 10 min at 95°C, followed by 40 cycles of 15 s at 95°C, 30 s at 50°C, and 60 s at 70°C, with a final cycle of 15 s at 95°C, 60 s at 60°C, 15 s at 95°C, and 15 s at 60°C.

### Library preparation and shallow shotgun metagenomic sequencing

To investigate bacteria, DNA extracted from infant stool samples was utilized for library preparation and shallow shotgun metagenomic sequencing. Libraries were prepared using the Nextera library preparation kit, adhering to the standard protocol (Illumina, San Diego, CA, USA). Samples were paired-end (PE) sequenced using NextSeq 500 (Illumina, San Diego, CA, USA). Around 26.94 Gbases were generated using 2 × 150 paired-end reads. Each sample yielded a median of 1.13 Gbases, which is very close to our intended target of 1 Gbase per fecal sample.

### DNA amplification and 18S ribosomal RNA gene amplicon sequencing

The eukaryotic 18S rRNA gene in DNA extracted from infant stool samples was amplified using the Comeau primers E572F: 5′-CYGCGGTAATTCCAGCTC-3′ and E1009R: 5′-AYGGTATCTRATCRTCTTYG-3′ [19]. The reaction included a peptide nucleic acid (PNA) blocking primer to reduce the amplification of mammalian sequences 5′-TCTTAATCATGGCCTCAGTT-3′ [20]. Amplification was performed in duplicate, with one reaction using undiluted DNA and one reaction using DNA diluted 1:10 in PCR water, according to previously described protocols [19]. Two mock community samples, including *Blastocystis, Candida*, and *Pichia*, were used as positive controls to assess the reliability of PCR amplification and sequencing and to estimate biases. E-Gels were used to visualize PCR products. DNA was quantified using an Invitrogen Qubit Fluorometer with Picogreen (Thermo Fisher Scientific, Waltham, MA, USA) and pooled at equal concentrations. PhiX was spiked in at 5%, and the resulting library was sequenced using MiSeq 500 Cycle Reagent Kit v2 (Illumina, San Diego, CA, USA) with a maximum read length of 2 × 250 bp.

### Bioinformatic analyses

#### Shotgun metagenomic data processing and quality filtering

After sequencing, reads were demultiplexed and quality controlled by the sequencing company. Additionally, we evaluated the quality of reads using FastQC v0.11.9 [21], followed by MultiQC v1.12 [22]. We filtered samples with a very low number of reads (<1 000 000 reads/sample) and used KneadData v0.10.0 to trim adapters, primers, and low-quality sequences and to remove human genome contamination. We also trimmed low-quality sequences and decontaminated reads from the human genome by detecting human-specific k-mers using Trimmomatic v0.33 [23] (parameters: PE -phred33, TRAILING:3, SLIDINGWINDOW: 4 : 30, MINLEN: 60 and HEADCROP:10) and BMTagger v3.101 [24], using the human genome reference database recommended in the KneadData pipeline. For taxonomic profiling of infants’ stool samples, we applied MetaPhlAn 4.0 [25] to map reads to the database of reference sequences (ChocoPhlAn vJan21) using bowtie2 v2.4.5 [26]. The average percentage of reads mapped to the database was 81.4% ± 8.97% (mean ± SD), and the average percentage of reads assigned to specific taxa was 49.4% ± 6.55% (mean ± SD) (Supplementary Fig. S1). For further analysis, we used the *metaphlan_to_phyloseq()* function from the *metaphlanToPhyloseq* package v0.2.0 [27] in R v4.3.3 [28] to convert the MetaPhlAn outputs into a format suitable for downstream statistical and ecological analyses. To denoise data and minimize sequence artifacts caused by sequencing errors [29], we performed the following steps: (i) removed all non-bacterial species-level genome bins (SGBs, as defined by MetaPhlAn4) (0.01% of all sequences, including unclassified SGBs at the Kingdom level), (ii) eliminated two outlier samples that had a quite substantially different community diversity relative to other samples, in terms of within-sample diversity as measured by Shannon diversity and/or between-sample dissimilarity as measured by non-metric multidimensional scaling on Bray–Curtis dissimilarities [30], (iii) removed two samples with fewer than 500 sequences, (iv) identified and filtered contaminating DNA from microbial communities, using the frequency method from the *decontam* package v1.22.0 [31], which detected 15 SGBs as contaminants based on their distribution relative to the DNA concentrations of the samples, (v) excluded rare SGBs with <1% abundance in the entire dataset (accounting for 6.3% of SGBs). In total, we identified 963 distinct bacterial SGBs across 126 samples with an average of 87 ± 41 SGB richness (mean ± SD) per sample (Supplementary Table S1.A). Considering MetaPhlAn’s results have already been normalized [25], we did not perform any further rarefaction or normalization.

To perform functional profiling of infants’ samples, we mapped reads against the UniRef90 protein databases derived from ChocoPhlAn species pangenomes using HUMAnN 3.0 [32]. This process involved using Bowtie2 v2.4.5 [26] and the DIAMOND aligner v2.1.11 [33]. We denoised data through the following steps: (a) removing unmapped reads that were not aligned to any reference gene (22.5% of total normalized pathway abundance) and unintegrated reads that were aligned to genes but not to any defined pathway (71.4% of total normalized pathway abundance), resulting in retaining 6.1% of the normalized community signal that was assigned to known MetaCyc pathways, (b) discarding one outlier sample with a very different composition based on Bray–Curtis dissimilarities, and (c) keeping only functional pathways that appeared at least three times in 10% of the samples, which filtered out 19% of pathways. As a result, we identified 371 different functional pathways associated with the gut bacteriome of rural infants across 129 samples, with an average of 241 ± 71 (mean ± SD) pathways per sample.

#### 18S rRNA gene sequencing data processing and quality filtering

For the 18S dataset, we applied the DADA2 pipeline [34] on adapter- and primer-free demultiplexed samples received from Microbiome Insights. Using the *DADA2* package v1.26.0 [34] in R, we denoised and filtered low-quality sequences, merged paired-end sequences, eliminated chimeric sequences, and identified amplicon sequence variants (ASVs). Then, we annotated the taxonomic identity of the ASVs by assigning them to species-level taxonomy using the Ribosomal Database Project (RDP) naive Bayesian classifier method implemented in the *DADA2* package with the dada2-formatted SILVA v132 rRNA database for eukaryotes [35] with the default parameter settings.

We removed the positive controls from the dataset after verifying the presence of mock communities in them. Then, we performed the following steps of quality verifying, filtering, and decontamination to minimize taxon overestimation and sequence artifacts caused by PCR and sequencing errors: (1) all ASVs were taxonomically classified as eukaryotes; (2) we removed five samples that had a very different composition from the others based on alpha or beta diversity; (3) we filtered out 10 samples that contained <1000 sequences; (4) we identified and removed nine ASVs as contaminants using the frequency method from the *decontam* R package; (5) we eliminated all the ASVs recognized as chloroplasts (plant families within the Chloroplastida class) or Metazoa (28.8% of ASVs; in total, we removed 73 Metazoan ASVs and 283 chloroplast. There were no ASVs assigned to mitochondria); and (6) we excluded rare ASVs with <10 reads (18.3% of ASVs). Overall, we identified 704 distinct eukaryotic ASVs across 121 samples with an average of 34 ± 27 ASV richness (mean ± SD) per sample (Supplementary Table S1.B). To assess fungal diversity and composition variation, we extracted fungi from the denoised eukaryote dataset and removed two samples that contained no fungal community and one sample that showed a very different composition based on Bray–Curtis dissimilarities. Before performing community-level diversity analyses, such as alpha and beta diversity, which are sensitive to sequencing depth, we used the variance-stabilizing transformation implemented in the *DESeq2* package v1.43.4 [36] in R. This approach helps address potential heteroscedasticity and overdispersion in samples while avoiding the loss of information through rarefaction [37].

### Statistical analyses

To identify the early-life composition and diversity of the gut microbiome in rural infants, we analysed denoised and normalized data from the stool samples collected from the first to the 18th month of age from 10 infants (P1–P10) from a rural district of Morelos, Mexico. We excluded infant P7 from our statistical analyses due to missing samples in the 13 to 18-month age group. To examine these rural infants’ gut microbiome variation and diversity, we studied gut bacteriome and eukaryome, using the metagenomic and 18S datasets, respectively. To ensure each level of the variables under study was adequately represented in the statistical and ecological analyses, we included only variables with adequate sample sizes that were neither too sparse nor too uniform in their sample distributions across variable levels (Supplementary Table S2). After each statistical analysis, we also conducted sensitivity analyses for the variables excluded from the selected models and compared the results using analysis of variance to confirm there were no statistically significant changes in the results by inclusion of these additional covariates.

We analysed taxonomic and functional alpha and beta diversity and dispersion to assess microbial community variation across study variables that had an adequate sample size among infants, including infant age group, mode of birth, sex, animal exposure, weaning age, and household size. For alpha diversity, we calculated the Shannon index using the *estimate_richness()* function of the *phyloseq* R package v1.50.0 [38]. For beta diversity, we used the *betadisper()* function from the *vegan* package v2.7.1 [39] to test for multivariate homogeneity of group dispersions, and we extracted average distances to centroids as a quantitative measure of microbial community dispersion. To address the longitudinal nature of the study, we used linear mixed-effects models (LMMs) [40] using the *lmer()* function from the *lme4* R package v1.1.37 [41] to evaluate variation in alpha diversity and dispersion across samples and incorporated infant identity as a random effect [model: . ~ variables + (1 | infant identity)], with parameter estimation via restricted maximum likelihood. Using the *performance* R package v0.15.0 [42], we also calculated marginal *R*^2^ (*R*^2^m; variance explained by fixed effects) and conditional *R*^2^ (*R*^2^c; variance explained by both fixed and random effects) to evaluate model fit. Residuals from initial LMMs were evaluated for normality using the Shapiro–Wilk test [43] and Q–Q plots. Model performance was evaluated using the Akaike Information Criterion (AIC) [44]. To rank models based on AICs for different variable combinations, we used the *dredge()* function from the *MuMIn* R package v1.46.0 [45]. To evaluate our hypotheses, we selected the model with the highest *R*^2^m and lowest AIC scores that included infant age, mode of birth, and the greatest number of other variables (sex, animal exposure, weaning age, and household size). Finally, for each of the microbial communities (bacteria, eukaryotes, and fungi), we reperformed LMMs based on the model ranking results and using the variable sets from the selected fitted models (Supplementary Tables S3 and S4). To evaluate variation in Shannon diversity and dispersion across variables, we performed post hoc pairwise comparisons of estimated marginal means (EMMs) using the *emmeans()* function from the *emmeans* R package v1.11.1 [46], applying the Kenward–Roger method for degrees-of-freedom approximation [47], with a 95% confidence interval (CI) included. Statistical significance of the pairwise group comparisons was assessed using the Wald test statistics. *P*-values were adjusted using Tukey’s Honestly Significant Difference method [48] to control for multiple comparisons. To evaluate how infant age and mode of birth influenced changes in alpha diversity over time, we calculated the *R*^2^m and *R*^2^c of the LMMs using age in months (considered as a continuous variable) separately for age [model: . ~ months + (1 | infant identity)] and mode of birth [model: . ~ months + mode of birth + (1 | infant identity)]. To examine the effects of study variables on microbial community taxonomic and functional composition variation, we conducted permutational multivariate analyses of variance (PERMANOVA) [49] using the *adonis2()* function within the *vegan* R package with 999 permutations on the community dissimilarity matrix (model: . ∼ mode of birth + weaning age + household size + animal exposure + sex + infant age group). To account for the longitudinal structure of the data, we used a permutation design that constrained permutations within, but not across, infant identities: *Plots(strata = infant identity, type = “none”)*, while maintaining the temporal order of samples: *Within(type = “series”)*.

To determine microbial taxonomic and functional differential abundance, we analysed normalized data using the *Maaslin2()* function in the *MaAsLin2* R package v1.16.0 [50] with default parameter settings, except for disabling standardization for all datasets and disabling data transformation for metagenomic data. This function applies a linear model to the transformed abundances of each group of samples and then uses Wald significance tests to identify significantly differentially abundant microbes between groups of the variables of interest, such as infant age group and mode of birth. For differential abundance analysis of microbial taxonomic profiles, we agglomerated bacterial data at the SGB level, while eukaryotic data were agglomerated at the genus level due to limitations in the SILVA database for species-level annotation of eukaryotes. We used the Benjamini–Hochberg (BH) false-discovery rate (FDR) method [51] to adjust the *P*-values.

To identify which microbial communities drove the variation in microbial composition as infants aged, we first combined samples that passed quality filtering steps in both the bacterial and eukaryotic datasets. Then we agglomerated each dataset to the genus level to account for heterogeneity across sequencing methods [52], normalized them to relative abundance, merged them, and performed principal coordinate analysis (PCoA) using the Bray–Curtis dissimilarities on combined genera (with relative abundance >0.01). Using the *envfit()* function of the *vegan* R package (with 999 permutations) and the BH FDR method [51] to adjust for multiple testing, we fitted the vectors of abundance for each genus as predictors of variation in microbial composition on the PCoA ordination. We assessed the goodness-of-fit values (*R*^2^) and their significance to determine which genera were significantly associated with variation within that age group.

To evaluate the relationships between infants’ gut bacterial and eukaryotic community compositions, we performed a Procrustes analysis [53] using the *procrustes()* function from the *vegan* package in R. This method assesses the correspondence between PCoA ordination coordinates of bacterial and eukaryotic communities across samples by rotating and scaling one ordination to fit the other one. Statistical significance and non-randomness of this concordance were evaluated using the *protest()* function with 999 permutations.

We performed ecological network analysis to determine inter-kingdom co-occurrences among microbial communities using the *NetCoMi* R package v.1.1.0 [54]. Due to limitations in the SILVA database for annotating eukaryotes at the species level, we agglomerated eukaryotic ASVs at the genus level and evaluated the co-occurrence of eukaryotic genera with bacterial species. We removed three outlier samples that had a substantially different community diversity compared to other samples based on Shannon diversity and/or a different composition based on Bray–Curtis dissimilarities. By applying the sparCC algorithm [55], we generated correlation-based networks to identify microbial co-occurrence, using the *netConstruct()* function and microbial absolute abundance (estimated based on the total microbial content quantified by qPCR, multiplied by microbial relative abundance). We assessed the statistical significance of the correlations using bootstrapped estimates with 1000 permutations. Significant correlations were defined as pairs of either bacteria–bacteria, eukaryotes–eukaryotes, or bacteria–eukaryotes with an absolute correlation co-efficient threshold >0.4 and BH-adjusted *P*-value <.001. To compare microbial associations across networks, we performed pairwise comparisons of networks based on their topological properties. To achieve this, we first calculated network topological properties for the whole network and for individual network nodes (i.e. microbial taxa) using the *netAnalyze()* function and the fast greedy clustering algorithm, which detects community structures by grouping taxa into clusters that are more connected [56]. Network global metrics included: (i) the number of nodes (i.e. the connected taxa), (ii) clustering coefficient (probability that the adjacent nodes of a node are connected, i.e. the probability that two microbial taxa that are both connected with a third taxon are also correlated with each other) [57], (iii) modularity (the degree of division of a network into clusters of co-occurring taxa) [58], (iv) positive edge percentage [the percentage of edges (microbial co-occurrences) with positive correlations to the total number of edges], (v) edge density (the ratio of the actual number of edges and the highest possible number of edges in a network, i.e. the proportion of microbial co-occurrences that are actually observed) [59], (vi) natural connectivity (the connectivity of a network as a measure of robustness) [60], (vii) vertex connectivity (the minimum number of nodes that must be removed from a network to disconnect it into separate clusters) [61], (viii) edge connectivity (the minimum number of links between taxa that must be removed from a network to disconnect it into separate clusters) [61], (ix) average dissimilarity (the mean of dissimilarity values), and (x) average path length (the mean of shortest paths, i.e. average minimum number of associations needed to connect pairs of microbial taxa). Network node-level metrics included: (a) degree (the number of adjacent links to a node), (b) betweenness centrality (the frequency with which a node acts as a bridge along the shortest paths between other nodes in a network; the taxa with the highest betweenness centrality might be identified as keystone microbial taxa) [62], (c) closeness centrality (the sum of reciprocals of shortest path lengths from a node to all others), and (d) eigenvector centrality (a measure of a node’s influence based on its connections) [63]. Then, we used the *netCompare()* function to perform pairwise comparisons of the networks’ topological properties using Jaccard’s index [64] and 5000 permutations. This function calculates *P*-values using a one-tailed test and adjusts them using the BH method. Finally, we conducted a differential network analysis using the *diffnet()* function to determine whether differential associations exist across networks, while controlling for multiple comparisons with the local FDR and the adaptive BH correction. Significance of differences was assessed using a posterior probability threshold of 0.5 and 1000 permutation tests.

## Results

### Taxonomic characterization of the infants’ gut microbiome in a rural setting

**Figure 1:**
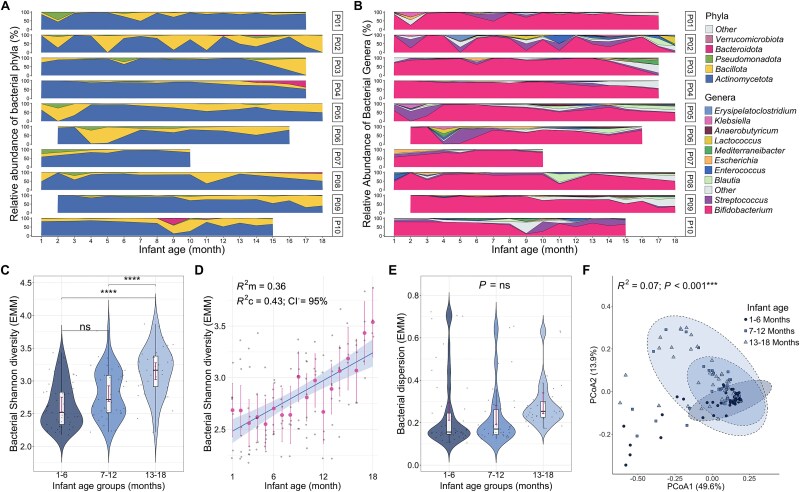
Gut bacterial community diversity and compositional variation from the first to the 18th month of age in infants from a longitudinal cohort in a rural area of Morelos, Mexico; temporal fluctuations of sample-level relative abundance of (a) phyla and (b) genera (agglomerated at the genus level) for each individual infant; (c) alpha diversity (Shannon index) at different age groups (LMM, Wald test, Tukey-adjusted); (d) temporal changes in alpha diversity (EMMs on LMM), error bars indicate a 95% CI; (e) dispersion at different age groups (LMM, Wald test, Tukey-adjusted); (f) PCoA on Bray–Curtis dissimilarities of community composition (PERMANOVA); each point represents one stool sample; points are distinguished by age group using different shapes; ellipses represent a 95% CI for each age group; significance levels for each variable: ^****^P < .0001, ^***^P < .001, ns: *P* > .05.

Profiling the gut microbial taxonomy of the rural infants in this cohort revealed a total of 8 bacterial phyla and over 150 genera. The five most abundant phyla were *Actinomycetota, Bacillota, Pseudomonadota, Bacteroidota,* and *Verrucomicrobiota* (Fig. 1a). The 10 most abundant bacterial genera included *Bifidobacterium, Streptococcus, Blautia, Enterococcus, Escherichia, Mediterraneibacter, Lactococcus, Klebsiella, Anaerobutyricum,* and *Erysipelatoclostridium*, with *Bifidobacterium* dominating across samples (Fig. 1b; also shown by age group and delivery mode in Supplementary Fig. S2A–D).

**Figure 2:**
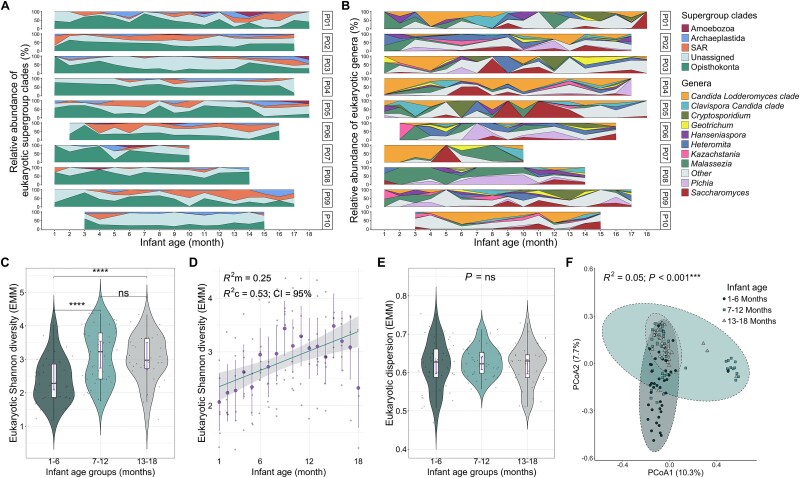
Gut eukaryotic community diversity and compositional variation from the first to the 18th month of age in infants from a longitudinal cohort in a rural area of Morelos, Mexico; temporal fluctuations of sample-level relative abundance of (a) supergroup clades and (b) genera (agglomerated at the genus level) for each individual infant; (c) alpha diversity (Shannon index) at different age groups (LMM, Wald test, Tukey-adjusted); (d) temporal changes in alpha diversity (EMMs on LMM), error bars indicate a 95% CI; (e) dispersion at different age groups (LMM, Wald test, Tukey-adjusted); (f) PCoA on Bray–Curtis dissimilarities of community composition (PERMANOVA); each point represents one stool sample; points are distinguished by age group using different shapes; ellipses represent a 95% CI for each age group; SAR: *Stramenopila, Alveolata*, and *Rhizaria*; significance levels for each variable: ^****^P < .0001, ^***^P < .001, ns: *P* > .05.

Using the SILVA v132 database, most microbial eukaryotic taxa classified at the phylum level corresponded to major supergroup-level clades. Our samples included phyla from four eukaryotic supergroup clades: *Opisthokonta*, SAR (*Stramenopila, Alveolata*, and *Rhizaria*), *Archaeplastida*, and *Amoebozoa* (Fig. 2a). These samples included over 100 microbial eukaryotic genera, including eight fungal genera (*Candida Lodderomyces* clade*, Malassezia, Saccharomyces, Pichia, Clavispora Candida* clade*, Hanseniaspora, Geotrichum,* and *Kazachstania*), one genus from the Apicomplexa order (*Cryptosporidium*), and one from the Cercozoa order (*Heteromita*) (Fig. 2b, also shown by age group and delivery mode in Supplementary Fig. S2E–H).

### Temporal dynamics of infants’ gut microbiome in a rural setting

Age influenced the gut microbiome of rural infants, resulting in distinct alpha diversity patterns in bacterial and eukaryotic communities. During the first 18 months of age, bacterial alpha diversity increased as infants grew older, especially after their first year (Shannon index mean ± SD 1–6 months: 2.62 ± 0.34, 7–12 months: 2.82 ± 0.37, 13–18 months: 3.15 ± 0.38; LMM; Wald test; Tukey’s pairwise comparisons 1–6 vs 13–18 months and 7–12 vs 13–18 months: adjusted *P* < .0001; Fig. 1c; Supplementary Table S3). In contrast, microbial eukaryotic alpha diversity peaked earlier in infancy, around the time of introducing complementary feeding (Shannon index mean ± SD 1–6 months: 2.4 ± 0.69, 7–12 months: 3.12 ± 0.8, 13–18 months: 3.15 ± 0.38; LMM; Wald test; Tukey’s pairwise comparisons 1–6 vs 7–12 months and 1–6 vs 13–18 months: adjusted *P* < .0001; Fig. 2c; Supplementary Table S3). Among eukaryotic communities, fungal alpha diversity remained stable as infants aged (Supplementary Fig. S3A and B; Supplementary Table S3).

Infant age explained 1.5 times more variance in both gut bacterial alpha diversity and community composition than in eukaryotes, but did not affect microbial homogeneity. Specifically, 36% of the monthly variation in infants’ gut bacterial alpha diversity was associated with their age, with an additional 7% variation explained by their identity (*R*^2^m = 0.36, *R*^2^c = 0.43; LMM, 95% CI; Fig. 1d). In comparison, 25% of the monthly eukaryotic community variation was explained by their age, quite similar to the 28% explained by their identity (*R*^2^m = 0.25, *R*^2^c = 0.53; LMM, 95% CI; Fig. 2d). Infant age had no significant impact on the homogeneity of microbial communities (Figs 1e and 2e, and  Supplementary  Fig.  S3C). Yet, it was among the strongest drivers of microbial community composition, accounting for 7% of the variation in bacterial composition and 5% in eukaryotic composition (bacteria: *R*^2^ = 0.07; eukaryotes: *R*^2^ = 0.05; fungi *R*^2^ = 0.05; PERMANOVA *P* < .001; Figs 1f and 2f, and  Supplementary  Fig.  S3D; Table 1).

**Table 1:** Main drivers of microbial community composition variation in infant stool samples from a rural setting.

	Bacteria			Eukaryotes			Fungi		
Variables	*R* ^ 2^	*F*	Pr(>*F*)	*R* ^ 2^	*F*	Pr(>*F*)	*R* ^ 2^	*F*	Pr(>*F*)
**Mode of birth**	0.01	1.85	0.001^***^	0.01	1.93	0.001^***^	<0.01	1.07	0.001^***^
**Sex**	0.02	3.56	0.001^***^	0.02	2.59	0.001^***^	0.01	1.29	0.001^***^
**Weaning age**	0.05	3.89	0.001^***^	0.05	3.46	0.001^***^	0.03	2.05	0.001^***^
**Household size**	<0.01	0.62	0.001^***^	0.02	2.53	0.001^***^	0.02	1.99	0.001^***^
**Animal exposure**	0.03	2.18	0.001^***^	0.05	3.6	0.001^***^	0.04	2.33	0.001^***^
**Infant age group**	0.07	4.99	0.001^***^	0.05	3.21	0.001^***^	0.05	3.39	0.001^***^

PERMANOVA based on Bray–Curtis dissimilarities determines the contribution of each variable to the variation in infants’ gut microbial composition.

**Figure 3:**
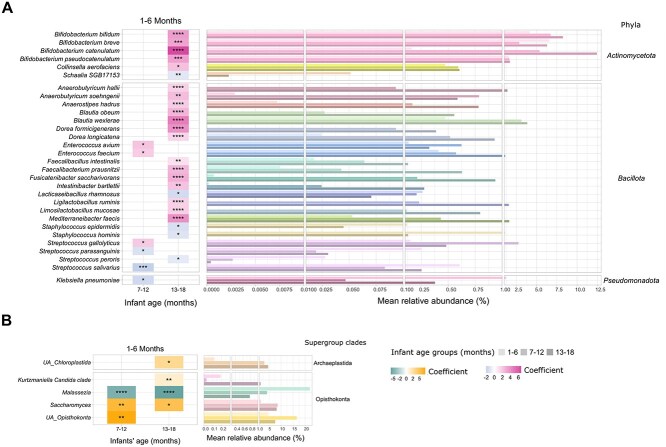
Gut microbial taxa associated with different age groups and their relative abundance from the first to the 18th month of age in infants from a longitudinal cohort in a rural area of Morelos, Mexico; differential abundance analysis illustrates the (a) bacterial taxa (phyla and species) and (b) eukaryotic taxa (supergroups and genera, rather than species, due to limitations of the database in identifying eukaryotes at the species level) that were significantly differentially more abundant across all age groups among rural infants (MaAsLin2, BH-adjusted *P* < .05); taxa with positive coefficients are associated with the reference age group, and taxa with negative coefficients are associated with the age group on the x-axis; barplots represent the mean relative abundance of the corresponding taxa; stacks sharing the same genus are grouped together; unidentified taxa are labeled with “*UA_*” followed by their corresponding higher taxonomic level.

**Figure 4:**
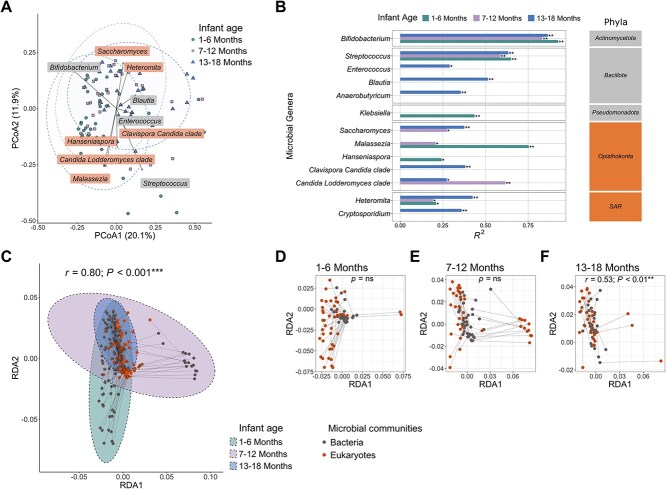
Correlations between gut microbial variation and age groups, as well as between bacterial and eukaryotic compositions, in each sample from the first to the 18th month of age in infants from a longitudinal cohort in a rural area of Morelos, Mexico; a and b: envfit analysis showing correlations between microbial genera PCoA ordination; (a) whole dataset: each point represents a stool sample, with shapes used to differentiate age groups; arrows represent genera (relative abundance >0.01) that were significantly associated with the ordination based on envfit (BH-adjusted *P* < .01); arrow direction indicates the gradient along which a genus increases, and arrow length reflects the strength of the correlation (*R*^2^); ellipses represent a 95% confidence interval (CI) for each age group; (b) age-group specific associations: for each age group, bars represent genera (relative abundance >0.01) significantly correlated with one or more age groups (BH-adjusted *P* < .05); bar length corresponds to the strength of the correlation (*R*^2^); c–f: Procrustes analysis illustrating the spatial concordance between PCoA ordination of gut bacterial and eukaryotic communities for the entire dataset (c) and stratified by age group (d: 1–6 months, e: 7–12 months, f: 13–18 months); each point represents one stool sample plotted twice: once for its bacterial community and once for its eukaryotic community; the line connecting each pair represents the Procrustes residual between bacterial and eukaryotic compositions within that sample; ellipses represent a 95% CI for each age group; SAR: *Stramenopila, Alveolata*, and *Rhizaria*; significance levels for each variable: ^***^P < .001, ^**^P < .01, ^*^P < .05, ns: *P* > .05.

The relative abundance of multiple microbial taxa changed significantly over time, with several gut bacterial and eukaryotic taxa displaying distinct temporal trends. Specifically, we noted differentially significant increases in 22 bacterial species (28 unique SGBs) from the *Actinomycetota* and *Bacillota* phyla and decreases in 8 species (11 unique SGBs) from the *Bacillota* and *Pseudomonadota* phyla as infants grew older. For example, the abundance of some *Bifidobacterium* species (*Bifidobacterium breve, Bifidobacterium catenulatum,* and *Bifidobacterium pseudocatenulatum*), *Blautia obeum, Faecalibacterium prausnitzii, Ligilactobacillus ruminis, Limosilactobacillus mucosae*, and *Mediterraneibacter faecis* differentially increased from both 1–6 and 7–12 months to 13–18 months, but not between 1–6 and 7–12 months. We also observed an increase in *Bifidobacterium bifidum* and *Blautia wexlerae* from 1–6 months to 13–18 months, whereas the increase from 1–6 to 7–12 months was not significant. *Enterococcus* species (*Enterococcus avium* and *Enterococcus faecium*) increased only between 1–6 and 7–12 months. Finally, *Dorea* species (*Dorea formicigenerans* and *Dorea longicatena*) showed a stepwise differential increase from 1–6 to 7–12 months and from 7–12 to 13–18 months. Conversely, there was a significant decline in the abundance of some other bacteria, including *Klebsiella pneumoniae* from 1–6 to 7–12 months, *Streptococcus peroris* from 1–6 to 13–18 months, and *Streptococcus salivarius* from 1–6 months to both 7–12 and 13–18 months (MaAsLin2 BH-adjusted *P* < .05; Fig. 3a and Supplementary Table S5.A). Among infants’ gut eukaryotic genera, *Malassezia* from the *Opisthokonta* supergroup showed a significant decrease from 1–6 to 7–12 months and from 7–12 to 13–18 months. In contrast, five eukaryotic genera from the *Archaeplastida* and *Opisthokonta* supergroups showed significant increases with age. These included the *Kurtzmaniella Candida* clade, which differentially increased from both 1–6 and 7–12 months to 13–18 months, and *Saccharomyces*, which increased from 1–6 months to both 7–12 and 13–18 months (MaAsLin2 BH-adjusted *P* < .05; Fig. 3b and Supplementary Table S5.B). Age-specific patterns in microbial abundance were strongly linked to shifts in gut microbial composition, with certain bacterial and eukaryotic genera displaying significant correlations across different developmental stages (envfit analysis of correlation between PCoA axes and variables, BH-adjusted *P-*values <.01; Fig. 4a; Supplementary Table S6.A). Changes in the relative abundance of the bacterial genera *Bifidobacterium* and *Streptococcus* and the eukaryotic genus *Heteromita* were linked to variation in microbial composition across all three age groups, likely reflecting gradual shifts in species abundance within these bacterial genera. Whereas the relative abundance of some other microbial genera was correlated with only one or two age groups, including (1) *Klebsiella* (bacteria) and *Hanseniaspora* (eukaryote) in 1–6 months, (2) *Anaerobutyricum, Blautia,* and *Enterococcus* (bacteria), and *C. Candida* clade and *Cryptosporidium* (eukaryote) in 13–18 months, (3) *Malassezia* (eukaryote) in 1–6 and 7–12 months, and (4) *C. Lodderomyces* clade and *Saccharomyces* (eukaryotes) in 7–12 and 13–18 months (envfit analysis of correlation between PCoA axes and variables, various BH-adjusted *P-*values; Fig. 4b; Supplementary Table S6.B). We also observed a significantly high correlation between the overall composition of infants’ gut bacteriome and eukaryome (Procrustes, *r* = 0.8, *P* < .001; Fig. 4c), particularly during 13–18 months (Procrustes; 1–6 months: *r* = 0.73, *P* = .23; 7–12 months: *r* = 0.68, *P* = .13; 13–18 months: *r* = 0.53, *P* < .01; Fig. 4d–f), suggesting that the developmental trajectories of these microbial communities align, specifically after 1 year of age.

### Drivers of variation in infants’ gut microbial community composition in a rural setting

In addition to age, animal exposure, mode of birth, sex, weaning age, and household size significantly contributed to the differences observed in gut microbial composition of these rural infants, collectively explaining 15%–20% of the variation (PERMANOVA *P* < .001; Table 1). Conversely, aside from infant age, none of the study variables had a significant impact on microbial taxonomic alpha diversity or dispersion (LMM, 95% CI; Supplementary Tables S3 and S4). Sensitivity analysis indicated no significant effects associated with other collected variables, including infant weight and height at various sampling times, presence of parasites, vaccination history, breastfeeding status, maternal education and occupation, housing conditions (except household size), water access and type, and energy sources. These variables were excluded from analysis because of insufficient sample sizes or overly uniform distributions across variable levels.

In this cohort, weaning age and animal exposure (types of animals with which infants were frequently in contact) emerged as strong drivers of gut microbial composition. They were both nearly as influential as the infant age on microbial composition variation, particularly on eukaryotic communities (composition variation explained by weaning age in bacteria: *R*^2^ = 0.05, eukaryotes: *R*^2^ = 0.05, and fungi: *R*^2^ = 0.03; composition variation explained by animal exposure in bacteria: *R*^2^ = 0.03, eukaryotes: *R*^2^ = 0.05, and fungi: *R*^2^ = 0.04; composition variation explained by infant age in bacteria: *R*^2^ = 0.07, eukaryotes: *R*^2^ = 0.05, and fungi: *R*^2^ = 0.05; PERMANOVA *P* < .001; Table 1).

Both infant sex and household size also had modest yet significant effects on the composition of their gut microbiomes. Sex explained ~0.01–0.02 of the variation in the overall gut microbial composition (bacteria: *R*^2^ = 0.02; eukaryotes: *R*^2^ = 0.02; fungi: *R*^2^ = 0.01; PERMANOVA *P* < .001; Table 1). Household size explained a smaller, but still significant, variation in gut microbial composition, particularly in eukaryotic and fungal communities (bacteria: *R*^2^ < 0.01; eukaryotes: *R*^2^ = 0.02; fungi: *R*^2^ = 0.02; PERMANOVA *P* < .001; Table 1).

**Figure 5:**
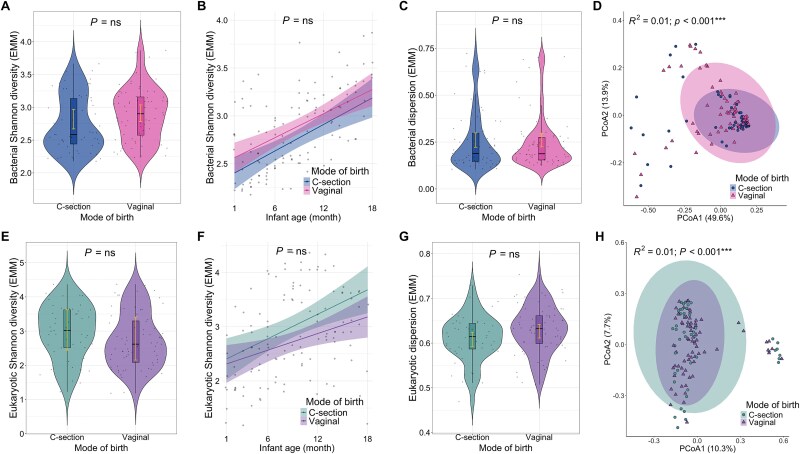
Gut microbial community diversity and compositional variation in response to mode of birth in infants (from the first to the 18th month of age) from a longitudinal cohort in a rural area of Morelos, Mexico; (a) bacterial and (e) eukaryotic alpha diversity (Shannon index) at each mode of birth (LMM, Wald test, Tukey-adjusted); temporal changes in (c) bacterial and (f) eukaryotic alpha diversity in response to mode of birth (EMMs on LMM); (c) bacterial and (g) eukaryotic dispersion in response to mode of birth (LMM, Wald test, Tukey-adjusted); PCoA on Bray–Curtis dissimilarities of (d) bacterial and (h) eukaryotic community composition (PERMANOVA); each point represents one stool sample; points are distinguished by mode of birth using different shapes; ellipses represent a 95% CI for each mode of birth; significance levels for each variable: ^***^P < .001, ns: *P* > .05.

Mode of birth also had a small but significant impact on microbial community composition, especially in bacterial and eukaryotic communities (*n* = 5 vaginally born vs 4 C-section infants; bacteria: *R*^2^ = 0.01; eukaryotes: *R*^2^ = 0.01; fungi: *R*^2^ < 0.01; PERMANOVA *P* < .001; Fig. 5d and h, and  Supplementary  Fig.  S3H; Table 1). Alpha diversity and community dispersion did not significantly differ between vaginally and C-section-delivered infants (Fig. 5a, c, e, and  g, and  Supplementary  Fig.  S3E and G; Supplementary Tables S3 and S4). Consequently, infants born through either delivery mode showed similar patterns of microbial alpha diversity over time (Fig. 5b and f, and  Supplementary  Fig.  S3F). Additionally, there was no significant impact of mode of birth on the abundance of gut microbial taxa (MaAsLin2).

Infant identity influenced taxonomic composition and alpha diversity in eukaryotic communities (Supplementary Fig. S2F and Fig.  2d) more than bacterial communities (Supplementary Fig. S2B and Fig.  1d). Specifically, it explained a substantially larger proportion of variance in eukaryotic alpha diversity (20%) than in bacteria (only 2%), highlighting stronger individual-level effects in eukaryotic communities (bacteria: *R*^2^m = 0.32 and *R*^2^c = 0.34; eukaryotes: *R*^2^m = 0.32 and *R*^2^c = 0.51; LMM, 95% CI; Supplementary Table S3). It also contributed 4% to fungal alpha diversity (*R*^2^m = 0.13 and *R*^2^c = 0.17; LMM, 95% CI; Supplementary Table S3). However, infant identity explained 6% of the variance in bacterial dispersion but did not affect eukaryotic or fungal dispersion (bacteria: *R*^2^m = 0.07 and *R*^2^c = 0.13; eukaryotes: *R*^2^m = 0.06 and *R*^2^c = 0.06; fungi: *R*^2^m = 0.05 and *R*^2^c = 0.05; LMM, 95% CI; Supplementary Table S4).

### Functional characterization of infants’ gut microbiome in a rural setting

**Figure 6:**
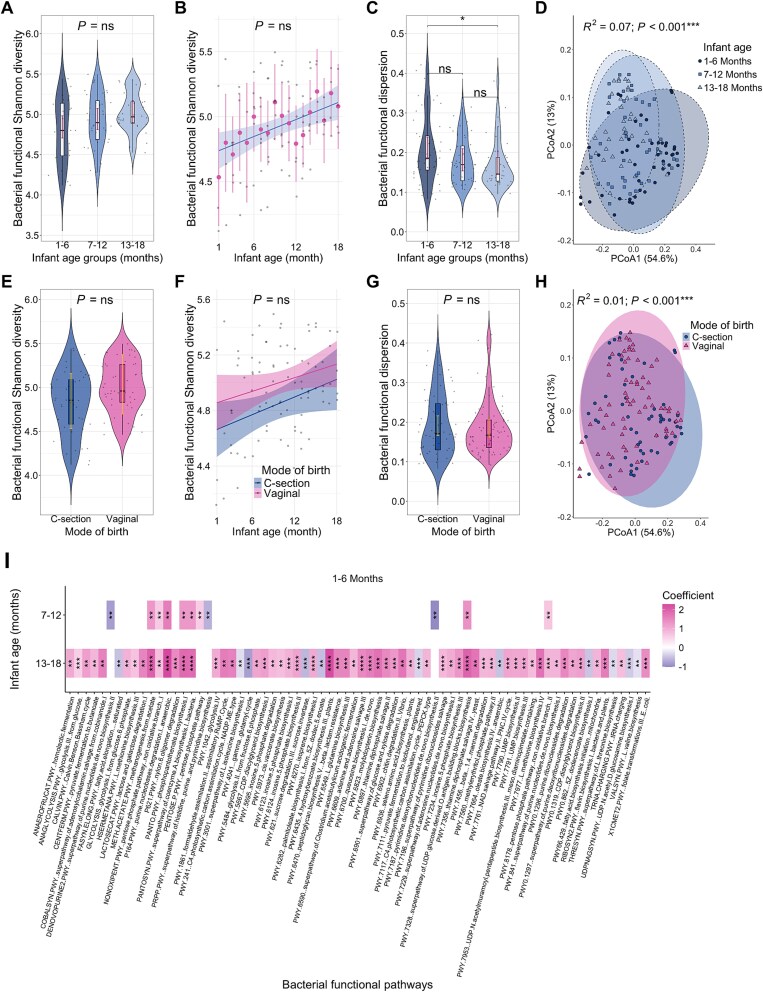
Gut bacterial functional variation from the first to the 18th month of age in infants from a longitudinal cohort in a rural area of Morelos, Mexico; functional alpha diversity (Shannon index) (a) for each age group and (e) in response to mode of birth (LMM, Wald test, Tukey-adjusted); temporal changes in functional alpha diversity (b) for each age group and (f) in response to mode of birth (EMMs on LMM); error bars in (b) indicate a 95% CI; functional dispersion (c) for each age group and (g) in response to mode of birth (LMM, Wald test, Tukey-adjusted); PCoA on Bray–Curtis dissimilarities of functional composition (d) for each age group and (h) in response to mode of birth (PERMANOVA); each point represents one stool sample; for each level of age group (d) and mode of birth (h), points are distinguished by different shapes, and ellipses indicate a 95% CI; (i) differential abundance analysis illustrates functional pathways that were significantly differentially more abundant across all age groups among rural infants (MaAsLin2, BH-adjusted *P* < .05); pathways with positive coefficients are associated with the reference age group, and those with negative coefficients are associated with the age group on the y-axis; significance levels for each variable: ^****^P < .0001, ^***^P < .001, ^**^P < .01, ^*^P < .05, ns: *P* > .05.

The variables under study collectively explained 17% of the variation in the functional composition of infants’ gut bacteria in this rural cohort (Table 2). However, bacterial functional alpha diversity and dispersion remained largely stable across variables during the first 18 months of life (Fig. 6a and b; Supplementary Table S3), with only a modest decrease in dispersion after the first year of age (average distance to centroid mean ± SD 1–6 months: 0.21 ± 0.09, 13–18: 0.17 ± 0.06; LMM; Wald test; Tukey’s pairwise comparisons 1–6 vs 13–18 months: adjusted *P* < .05; Fig. 6c; Supplementary Table S4). Consistent with our gut bacterial taxonomic findings, infant age was a significant driver of the bacterial metabolic pathway composition, accounting for 7% of the variation (*R*^2^ = 0.07; PERMANOVA *P* < .001; Fig. 6d; Table 2). Although mode of birth did not affect functional alpha diversity or dispersion (Fig. 6e–g; Supplementary Tables S3 and S4), it had a small but significant effect on overall gut bacterial functional composition (*R*^2^ = 0.01; PERMANOVA *P* < .001; Fig. 6h; Table 2), alongside other variables that also showed small yet statistically significant impacts (*R*^2^ = 0.01–0.03; PERMANOVA *P* < .001; Table 2).

**Table 2:** Main drivers of microbial functional variation in infant stool samples from a rural setting in Morelos, Mexico.

Variables	*R* ^ 2^	*F*	Pr(>*F*)
**Mode of birth**	0.01	1.69	0.001^***^
**Sex**	0.01	1.99	0.001^***^
**Animal exposure**	0.02	1.35	0.001^***^
**Weaning age**	0.02	1.47	0.001^***^
**Household size**	0.03	4.74	0.001^***^
**Infant age group**	0.07	5.12	0.001^***^

PERMANOVA based on Bray–Curtis dissimilarities determines the contribution of each variable in the infants’ gut microbial functional variation.

During the first 18 months of age, rural infants showed significant shifts in their gut bacterial metabolic potential. Among 371 identified bacterial metabolic pathways (see most abundant pathways in Supplementary Fig. S2I–L), 71 pathways exhibited a significant increase, and 25 pathways showed a significant decrease in differential abundance. This led to an increase in the differential abundance of 19.1% of pathways, while 6.7% showed a decrease, especially between the 1–6 and 13–18-month age groups (MaAsLin2; various BH-adjusted *P-*values; Fig. 6i and Supplementary Table S5.C). Overall, there were both increases and decreases across biosynthetic and catabolic pathways. The abundance of most bacterial degradation pathways, including those for starch degradation and nucleotide degradation, increased significantly with age. However, some carbohydrate degradation pathways showed a decline in abundance as infants grew older, including (i) 1–6 vs 7–12 months: sucrose degradation II (sucrose synthase) pathway, and (ii) 1–6 vs 13–18 months: sucrose degradation III (sucrose invertase), sucrose degradation IV (sucrose phosphorylase), stachyose degradation, and D-galactose degradation I (Leloir) pathways. Intriguingly, the abundance of the lactose and galactose degradation I pathway and the glucose and xylose degradation superpathway increased from 1–6 to 13–18 months. Beyond catabolic pathway trends, the abundance of formaldehyde assimilation II (RuMP Cycle), C4-photosynthetic carbon assimilation cycles (NADP-ME and PEPCK types), and all salvage pathways were consistently upregulated between 1–6 and 13–18 months of age (MaAsLin2, various BH-adjusted *P-*values; Fig. 6i; for the full list of shifts in metabolic pathways, see Supplementary Table S5.C). We observed no significant difference in the abundance of gut bacterial functional pathways between vaginal and C-section infants, indicating similar functional profiles across delivery modes.

### Shifts in gut microbial co-occurrence patterns over time and in response to birth mode in infants from a rural setting

**Figure 7:**
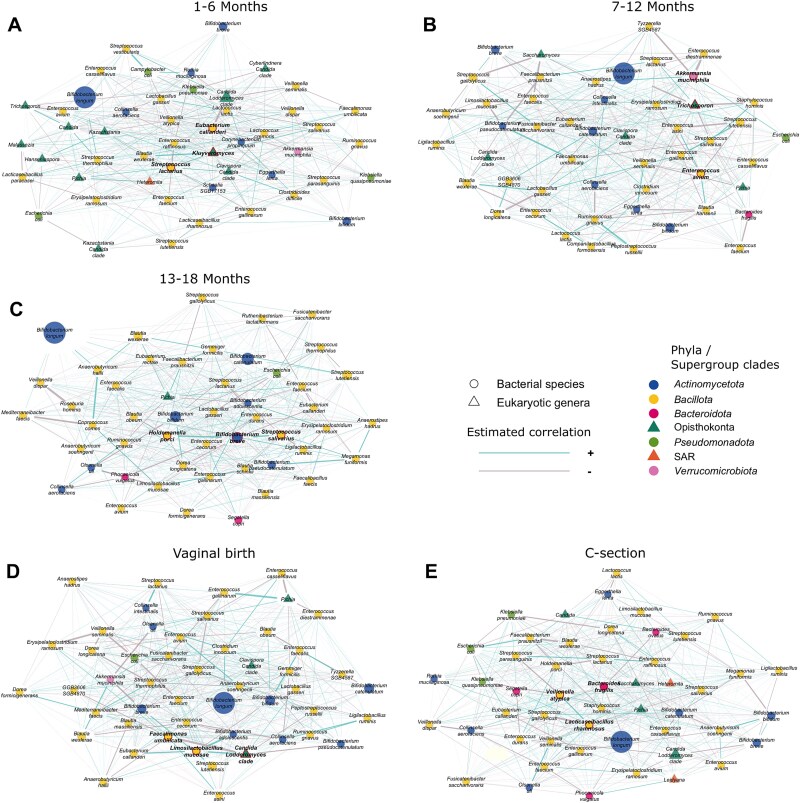
Inter-kingdom co-occurrence network analysis of gut bacterial and eukaryotic taxa present in stool samples from the first to the 18th month of age in infants from a longitudinal cohort in a rural area of Morelos, Mexico; microbial co-occurrence networks for each age group, (a) 1–6 months, (b) 7–12 months, and (c) 13–18 months, and in response to mode of birth, (d) vaginal birth and (e) C-section birth; each node represents one microbial taxon; node shape indicates the kingdom to which that genus belongs (triangle for eukaryotes, circle for bacteria); node size corresponds to the sum of normalized counts of microbes based on absolute abundance; node color shows the microbial phylum; only nodes with absolute correlations >0.4 and BH-adjusted *P* < .001 are represented, as determined by the SparCC algorithm; nodes with highlighted borders and bold labels signify network hubs; color of edges linking two nodes represent the direction of associations (positive or and negative correlations); edge width indicates the correlation strength; SAR: *Stramenopila, Alveolata*, and *Rhizaria*.

Gut microbial co-occurrence networks involving bacteria and eukaryotes evolved as infants grew older, showing a reduction in taxonomic diversity after the first year of life. During the first 6 months, we observed several taxa from four bacterial phyla (*Actinomycetota, Bacillota, Pseudomonadota*, and *Verrucomicrobiota*), and two eukaryotic supergroups (*Opisthokonta* and SAR) co-occurring. *Heteromita*, which was also among the age-specific eukaryotic genera (Fig. 4b; Supplementary Table S6.B), was the only member from the SAR supergroup observed in this network, and its abundance was positively associated with genera from the other present eukaryotic supergroup, *Opisthokonta* (including *Pichia* and *C. Lodderomyces* clade). By 7–12 months, *Bacteroides fragilis* from the bacterial phylum *Bacteroidota* was also detected, showing a negative association with several *Bacillota* species (including *E. faecium, Peptostreptococcus russellii*, and *S. salivarius*). However, no members of SAR were among the co-occurring taxa at this stage. Finally, between 13 and 18 months, the taxonomic diversity of co-occurring taxa declined and was restricted to four bacterial phyla (*Actinomycetota, Bacillota, Bacteroidota,* and *Pseudomonadota*) and the *Opisthokonta* eukaryotic supergroup (|correlation coefficient| > 0.4, BH-adjusted *P* < .001; Fig. 7a–c; Supplementary Table S7.A).

The taxonomic composition of microbial taxa that showed the strongest connections and emerged as network hubs notably changed during the first 18 months of age. The three network hubs included both bacteria and eukaryotes during the first year but were restricted to only bacteria after 12 months. Several bacterial species from *Bacillota* played a key role across all three age groups, and some eukaryotes from *Opisthokonta* also appeared as hub taxa during the first year, whereas all hubs in the 13–18-month age group were bacteria. Accordingly, hub taxa shifted from *Eubacterium callanderi, Streptococcus lactarius (Bacillota)*, and *Kluyveromyces* (*Opisthokonta*) at 1–6 months, to *Eubacterium avium (Bacillota), Akkermansia muciniphila (Verrucomicrobiota)*, and *Trichosporon* (*Opisthokonta*) at 7–12 months, and to *B. breve (Actinomycetota), Holdemanella porci*, and *S. salivarius (Bacillota)* at 13–18 months (|correlation coefficient| > 0.4, BH-adjusted *P* < .001; Fig. 7a–c; Supplementary Table S7.A).

Comparing network properties also confirmed significant age-related shifts in the structure of microbial co-occurrence patterns after 1 year of age. Although network global (like modularity) and node-level metrics (average centrality values) did not differ significantly across age groups, there were significant differences in the patterns of which microbial taxa were highly connected (degree of connectivity) and which taxa were highly accessible to the rest of the network (closeness centrality). Consequently, the most central taxa varied between the 1–6- and 13–18-month networks (Jaccard index for all significant metrics = 0.14, top-node threshold = 0.75, BH-adjusted *P* < .05; Supplementary Table S8.A). This suggests substantial turnover in the identity of central taxa despite similar average centrality values. In contrast, the 7–12-month network was not significantly different from the 1–6- and 13–18-month networks (Supplementary Table S8.A), indicating that the intermediate stage may represent a transitional network configuration sharing features with both earlier and later time points. We also observed a few qualitative shifts in individual associations; e.g. *Eggerthella lenta* and *S. lactarius* were negatively associated in the 1–6-month network but positively associated in the 7–12-month network, whereas *B. pseudocatenulatum* and *L. mucosae, Collinsella aerofaciens* and *Ruminococcus gnavus*, and *Anaerobutyricum soehngenii* and *Enterococcus faecalis* were negatively associated in the 7–12-month network but positively associated in the 13–18-month network (Supplementary Table S7.A). However, these differences were not statistically significant in the differential network analysis. This suggests that age-related differences between microbial co-occurrence networks primarily reflect changes in overall community structure and centrality patterns rather than shifts in specific taxon-taxon associations.

Mode of birth also influenced the infants’ gut microbial co-occurrence patterns, particularly in terms of central taxa and the overall network structure. Several co-occurring taxa in the gut of both C-section and vaginally delivered infants belonged to three bacterial taxa (*Actinomycetota, Bacillota*, and *Pseudomonadota*) and one eukaryotic supergroup (*Opisthokonta*). Vaginally born infants also showed taxa from the bacterial phylum *Verrucomicrobiota* in their gut microbial networks, whereas C-section infants exhibited taxa from the bacterial phylum *Bacteroidota* and the eukaryotic supergroup SAR, which co-occurred with other taxa in their gut. Hub microbial taxa in the gut of infants born through both modes of birth consisted of two bacterial species from the *Bacillota* phylum (vaginal delivery: *Faecalimonas umbilicata* and *L. mucosae*; C-section delivery: *Veillonella atypica* and *Lacticaseibacillus rhamnosus*), as well as *C. Lodderomyces* clade (*Opisthokonta*) in vaginally born infants, likely reflecting transmission through vaginal, breast milk, or skin contact [65, 66], and *B. fragilis* (*Bacteroidota*) in C-section infants (|correlation coefficient| > 0.4, BH-adjusted *P* < .001; Fig. 7d and e; Supplementary Table S7.B). Although global network metrics and average centrality values did not differ significantly between C-section and vaginally born infants, there were significant differences in the patterns of which taxa were highly connected (degree of connectivity), highly accessible to the rest of the network (closeness centrality), and strongly linked to keystone taxa and influential clusters (eigenvector centrality). Consequently, the most central taxa (with a high degree, closeness, or eigenvector centrality) varied between the C-section and vaginally born groups (Jaccard index for all significant metrics = 0.05, top-node threshold = 0.75, BH-adjusted *P* < .01; Supplementary Table S8.B), indicating substantial delivery-mode–specific turnover in the identity of central taxa. Therefore, the overall network structures differed between C-section and vaginally delivered infants. However, the differential network analysis detected no significant differences in individual pairwise associations. This indicates that, like age-related differences, birth-mode differences in gut co-occurrence networks were mainly driven by changes in community structure and key taxa rather than by significant changes in specific taxon-taxon associations.

## Discussion

The development of the gut microbiome follows ecological succession principles, where microbial colonization, interactions, and responses to environmental changes shape its composition over time [67, 68]. Like other natural ecosystems, the establishment of microbial species in the gut is influenced by factors such as competition, priority effects, and environmental filtering, all of which play a critical role in early-life microbiome development. Our findings align with previous research showing that gut microbiome maturation is not a simple linear inheritance process, but rather reflects a process of microbial community assembly driven by deterministic forces imposed by host development and environmental exposures. This process represents an adaptive response to environmental exposures, host traits, and developmental stages [69, 70].

The early-life gut microbiome of rural infants undergoes dynamic compositional and functional changes shaped by environmental exposures and host development. This pattern is consistent with a predominantly deterministic assembly process, in which host maturation and associated environmental changes impose strong selective pressures on microbial community composition. Our findings indicate that the gut microbiome of rural infants in early life experienced significant compositional shifts characterized by increased bacterial and eukaryotic diversity over time, with distinct maturation patterns. For example, infant age impacted the compositional variation and alpha diversity of their gut bacterial communities more than those of their eukaryotic communities. Additionally, the eukaryotic alpha diversity increased earlier in infancy, followed by a significant increase in bacterial diversity as infants grew older. However, fungal diversity was more stable over time. This finding illustrates divergent microbial trajectories that correspond to previous studies on both rural and urban infants. These studies have shown that although bacterial diversity generally increases with age, fungal diversity either remains stable [71–73] or decreases [74, 75] during the first year of life.

Despite a substantial increase in early-life gut bacterial taxonomic alpha diversity and no change in dispersion, their functional alpha diversity remained unchanged, although their functional pathways became less dispersed over time. This suggests that even as their taxonomic diversity shifts, core metabolic functions tend to remain stable. This observation may imply that the infant gut microbiome follows a model of functional redundancy, in which various bacterial taxa contribute to the same metabolic pathways, as proposed previously [76]. Furthermore, shifts in the abundance of bacterial biosynthetic and catabolic pathways suggest functional specialization as the microbiota matures. Even though environmental factors contributed modestly to functional variation, age remained the dominant factor, highlighting the essential role of host development in shaping microbial functions. These adaptations may reflect microbial responses to the nutritional demands of later infancy and are consistent with findings linking dietary transitions, such as the introduction of solid foods, to functional changes in the gut ecosystem [68, 77].

As the infants grew older, their gut bacterial and eukaryotic community compositions became more closely linked, especially after the first year of age, suggesting increased coordination or interactions at the community level. Our network analysis demonstrated that the shift in inter-kingdom network composition within the gut microbiome of rural infants, characterized by a decrease in eukaryotes and an increase in bacterial co-occurrence after the first year, was primarily driven by changes in the overall structure and connectivity of microbial communities, rather than associations among specific microbial taxa. This pattern is consistent with microbiome maturation, reflecting increased ecological structuring and stability of the community, in line with the process of selective microbial succession [78].

From an ecological perspective, our findings are consistent with principles of microbial succession, whereby early colonizers are progressively replaced or complemented by taxa adapted to evolving environmental conditions within the host. This process appears to be driven by niche-based selection, as shifts in diet and environmental exposures alter substrate availability and ecological constraints, promoting the expansion of taxa with distinct metabolic capabilities. The observed restructuring of inter-kingdom networks further suggests increasing community organization and potential niche partitioning over time. Together, these patterns support a model of early-life microbiome assembly as a dynamic and non-random process shaped by both deterministic ecological forces and changing host-associated environments.

Our findings indicated age-specific changes in the abundance of several bacterial and eukaryotic taxa. Specifically, we observed a decline in certain *Streptococcus* species (*S. peroris* and *S. salivarius*; known as early gut colonizers in infant gut), alongside an increase in *Bifidobacterium* species as infants grew older. While *Bifidobacterium* remained highly abundant throughout the study period, the observed changes primarily reflected species-level turnover rather than an overall increase in abundance. In particular, we observed increases in *B. breve* and *B. bifidum*, which are considered transitional or later-colonizing *Bifidobacterium* species. This pattern is consistent with niche differentiation within the genus, where closely related taxa occupy distinct metabolic niches as substrate availability shifts during dietary diversification. Although *Bifidobacterium* dominance in early life is strongly supported by breastfeeding through the provision of human milk oligosaccharides, these processes are most prominent in the early postnatal period [79, 80]. Given that our cohort already exhibited high *Bifidobacterium* abundance at the earliest time points, the changes observed between 1 and 18 months are unlikely to reflect initial colonization dynamics driven by breastfeeding alone.

Weaning age was another driver of gut microbial composition and functional pathways. This finding is consistent with previous research showing that the introduction of solid foods leads to significant shifts in infants’ gut microbial composition and function [10]. Certain age-related gut microbial changes observed in infants from this rural setting have been linked to dietary diversification following the introduction of complementary foods. These include increases in several *Bifidobacterium* species, *including B. bifidum and B. breve* [81, 82], and other bacterial species from the *Bacillota* phylum, such as *Blautia, Dorea, Faecalibacterium*, and *Mediterraneibacter* [10, 83], as well as a decline in *Malassezia* shifting toward *Saccharomyces* in eukaryotes [74, 84], after 6 months of age. In contrast to many urban cohorts [1, 85], where *Bifidobacterium* often peaks during exclusive breastfeeding and then declines around 6 months of age, we found that *Bifidobacterium* abundance remained high, alongside species-level diversification (*B. pseudocatenulatum, B. catenulatum, B. breve*, and *B. bifidum*). Our findings align with studies in rural areas, which have reported a sustained prevalence of *Bifidobacterium* communities after 6 months of age, as complementary foods are introduced and dietary complexity increases. A large cohort of 6 to 11-month-old rural Kenyan infants who received complementary foods in addition to breast milk showed a very high prevalence of *Bifidobacterium longum* subsp. infantis *(B. infantis)*, followed by *B. breve, B. bifidum*, and *Bifidobacterium kashiwanohense* [81]*.* Another large cohort of a peri-urban community in Bangladesh reported an increase in a transitional *B. longum* clade following the introduction of complementary food, which contains enzymes for both milk- and solid-food substrates [82].

In addition to weaning age, other early-life environmental factors, including animal exposure, household size, and mode of birth, were also key factors in shaping infants’ gut microbiomes in this rural setting. Previous ecological studies comparing rural and urban populations indicate that, in rural environments, infants are exposed to a different array of microbial sources (e.g. soil, animals, plants, and arthropods) and often have more frequent contact with domestic and wild animals [86, 87]. They are also influenced by differences in the microbial composition of mothers’ milk [3], earlier introduction of solid foods, larger household size, and dietary patterns with higher fiber intake [86, 88]. In our cohort, these exposures occurred in a rural community where households typically comprised three to six people, and access to piped water and energy sources was variable; all infants were breastfed, many infants had regular contact with pets, barn animals, cockroaches, and rodents, and solid foods were introduced at 3–5 months (see Supplementary Table S2) rather than the 6 months recommended by the World Health Organization [89].

The effect of mode of birth on microbial community composition was small, yet significant. However, compared with previous findings in both rural and urban settings [6, 8, 90, 91], these effects were relatively modest and less pronounced in our cohort, as alpha diversity and dispersion remained unchanged between C-section and vaginally born infants. This indicates that, in this rural setting, mode of birth showed only a limited influence on overall microbial diversity and community composition. A recent rural study has shown limited long-term effects of mode of birth on infants’ gut microbiome, associating the early colonization of community members such as *Bacteroides, Bifidobacteria,* and *Escherichia coli* to environmental exposure and contact with older siblings carrying these bacteria [92]. Additionally, the mode of birth significantly influenced the architecture of microbial associations, suggesting that mode of birth impacts not only the initial microbial colonization, but also the subsequent co-occurrence of community structures.

Overall, our results align with the notion that environmental exposures occurring later in infancy may play a more substantial role in shaping microbial composition and functionality [69, 70]. These findings emphasize the complexity of microbial colonization, where early birth conditions significantly shape bacterial taxonomic composition, while later environmental exposures remain among the main drivers of infants’ gut microbial community composition and diversity.

Our study offers insights into the early-life gut microbiome and highlights several key directions for further research, while also being subject to methodological and sampling limitations. First, despite the longitudinal and numerous sampling events per infant over 18 months, our study is limited by a small sample size, the lack of longitudinal health and dietary monitoring, and the fact that all infants were exclusively breastfed, which may introduce selection bias. Future studies should prioritize integrating detailed longitudinal health and dietary data to better resolve the relative contributions of breastfeeding, complementary feeding, and environmental exposures to microbiome assembly. Several collected variables were excluded from analysis because of sparse or highly unbalanced data, and for the variables retained, although some effects reached significance, the associated R^2^ values, though statistically significant, were generally modest. Expanding the cohort to include a larger sample size, more diverse feeding practices, and longitudinal maternal sampling would enhance the generalizability and explanatory power of our findings. Second, although we employed benchmark bioinformatic methods and widely recognized standard pipelines for our metagenomic analysis [25, 32], alternative methodological approaches could yield slightly different results. For instance, future evaluations of reference databases will be crucial to assess the impact of analysis and annotation methods on data interpretation. Third, previous studies show that low sequencing depth reduces detection sensitivity and underestimates diversity, potentially limiting the identification of low-abundance and rare taxa [93, 94]. To mitigate these biases and improve the reliability of our analysis, we excluded low-abundance taxa, a strategy that has been shown to increase the reproducibility of microbial analysis with minimal impact on the dominant community structure [95]. A multiapproach strategy combining metagenomic sequencing with culture-based techniques could provide a more comprehensive view of early-life microbial communities, particularly for low-abundance taxa that may be overlooked in shotgun sequencing [96, 97]. Gaining a deeper understanding of these microbial shifts and their functional implications may provide critical insights for microbiome-targeted interventions to support infant health and development.

In conclusion, our study offers valuable insights into the early-life gut microbiome of rural infants, revealing dynamic taxonomic and functional shifts influenced by aging, mode of birth, and environmental exposures. Even though bacterial and eukaryotic diversity increased with age, fungal diversity remained stable, suggesting divergent colonization trajectories. Our study further highlights the complexity of early microbial colonization and the role of functional redundancy in maintaining metabolic stability. Compared to urban infants, rural infants are often exposed to different microbial sources. This may facilitate the dispersal of microbes with higher fitness to colonize and persist in the gut, resulting in greater microbial diversity compared to urban infants and shaping distinct gut microbial succession patterns and inter-kingdom interactions [3, 86, 87, 92, 98, 99]. We suggest this is why environmental factors may play a stronger role than mode of birth in shaping distinct gut microbial succession patterns, as well as cross- and inter-kingdom interactions. Given the distinct environmental exposures of rural infants, further research should explore how traditional dietary habits and weaning practices contribute to microbial diversity and functionality. Comparative studies with urban cohorts, ideally from the same country or region, will be essential for understanding the influences of host, microbial, environmental, and social factors across gradients of human urbanization. Ultimately, broadening the scope of research to include diverse global populations will not only deepen our understanding of the early-life microbiome but also promote equitable health interventions that address the unique microbial needs of populations across different environments and cultures.

## Supplementary Material

Supplementary_material_wrag124

## Data Availability

Availability of data and materials. We have deposited the raw sequences at the NCBI Sequence Read Archive (SRA accession number PRJNA1258733). Our scripts to perform the current study analyses are available in the following GitHub repository: github.com/monaparizadeh/EarlyLifeGut_xoxo.
