# Supplementary material for: Impact of maternal, infant, and household factors on early-life gut microbiome development in a rural setting
Source: ISME J. 2026 May 13;20(1):wrag124. doi: 10.1093/ismejo/wrag124 (PMC13317942; doi:10.1093/ismejo/wrag124)
Supplement: Supplementary_material_wrag124 [file supplementary_material_wrag124.zip › Supplementary_Material _Parizadeh_etal.pdf]

Impact of Maternal, Infant, and Household Factors on Early-life Gut Microbiome Development in a Rural Setting

Parizadeh, M., et al.

*Corresponding author's email: marie.arrieta@ucalgary.ca

This file includes supplementary figures S1-S3 and accompanying captions for supplementary tables S1-S8. The supplementary tables are available in the file named "Supplementary_Tables_Parizadeh_etal".

Supplementary Figures

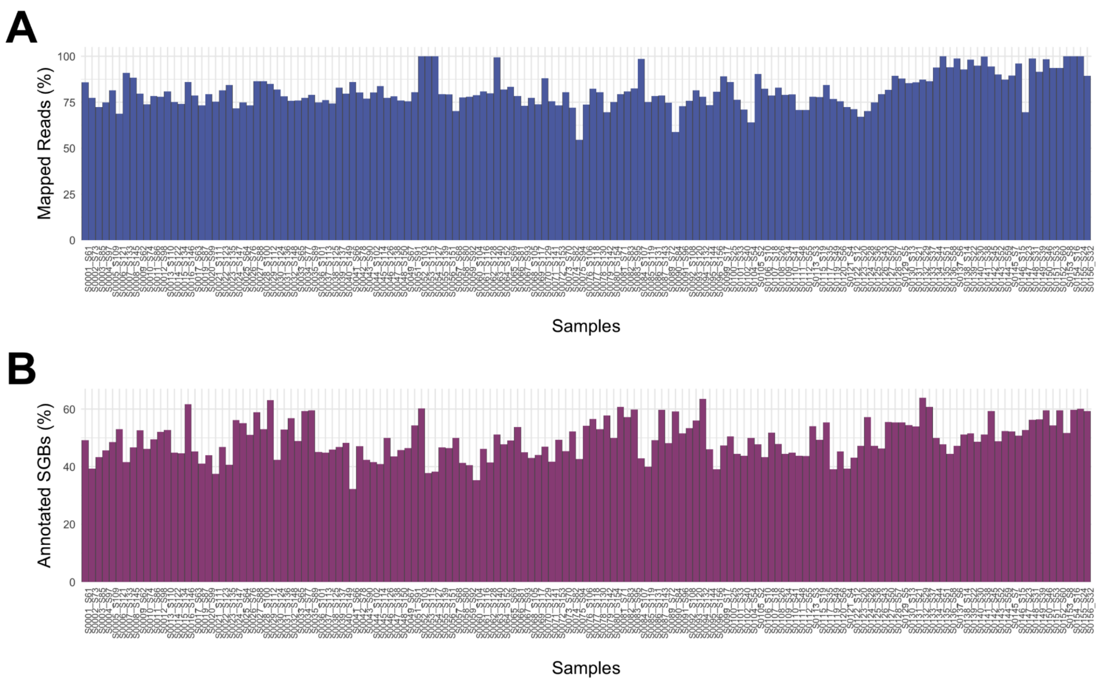

Figure S1. Taxonomic profiling of infants' stool samples, using MetaPhlAn 4.0 to map reads to the database of reference sequences (ChocoPhlAn vJan21). Percentage of reads mapped to the database per sample (A) and annotated SGBs per sample (B).

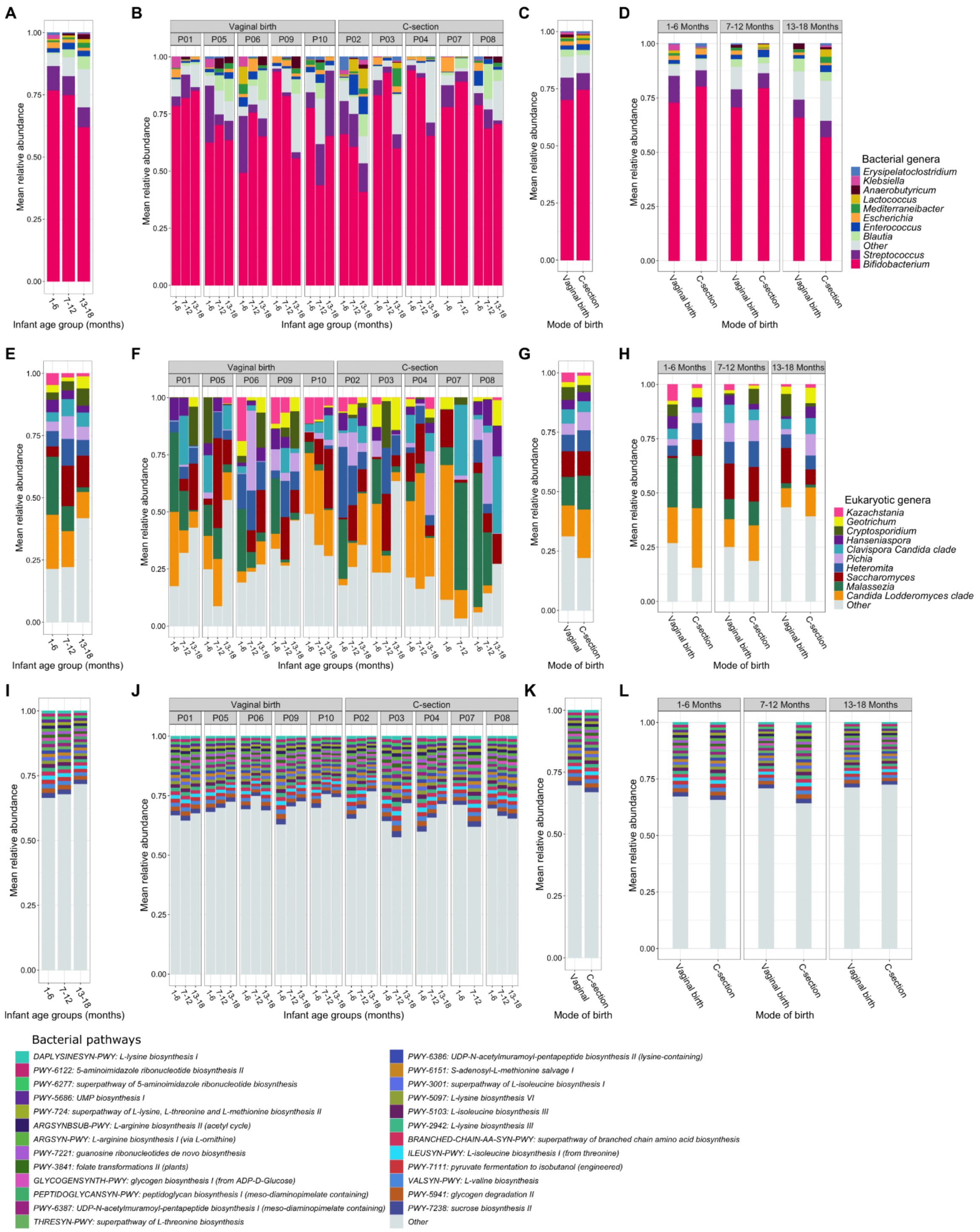

Figure S2. Mean relative abundance of bacterial (A-D) and eukaryotic (E-H) taxonomic communities and bacterial functional pathways (I-L) across stool samples of infants from a longitudinal cohort in a rural region of Morelos, Mexico, shown by age group (A, E, and I), each individual infant at different age groups (B, F, and J), delivery mode (C, G, and K), and delivery mode at different age groups (D, H, and L).

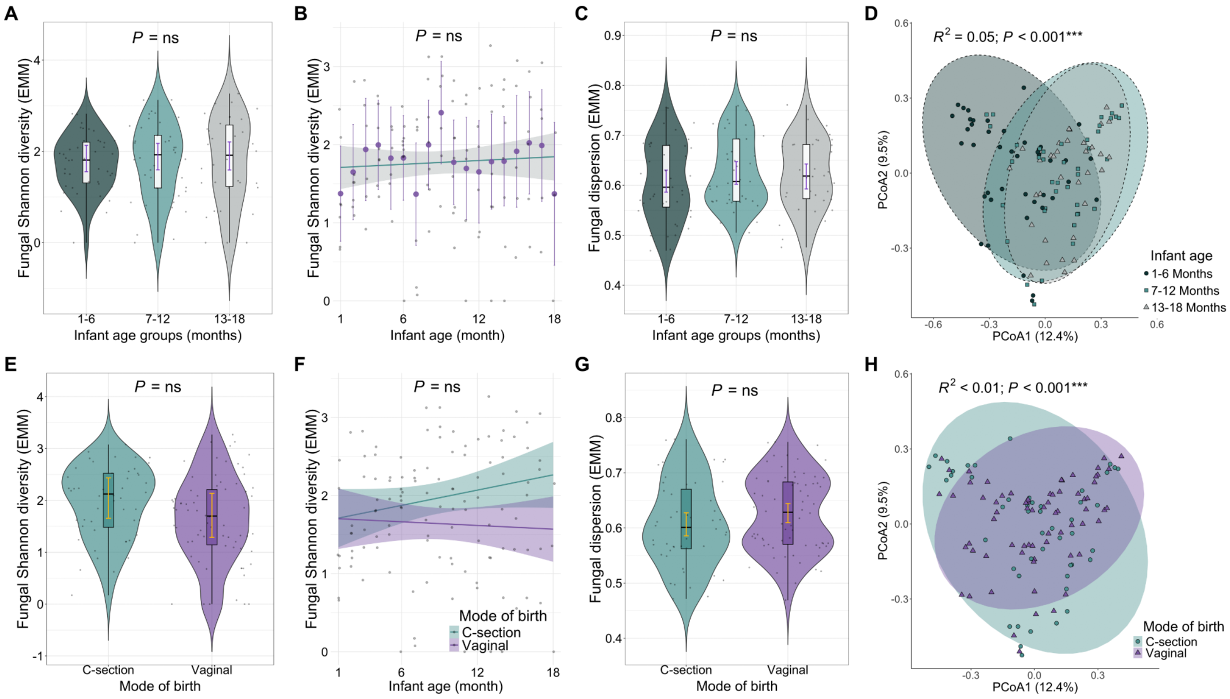

Figure S3. Gut fungal community diversity and compositional variation from the first to the 18th month of age in infants from a longitudinal cohort in a rural area of Morelos, Mexico. (A) Alpha diversity (Shannon Index) (A) for each age group and (E) in response to mode of birth (LMM, Wald test, Tukey-adjusted); temporal changes in alpha diversity (B) for each age group and (F) in response to mode of birth (EMMs on LMM); error bars in (B) indicate a 95% CI; dispersion (C) for each age groups and (G) in response to mode of birth (LMM, Wald test, Tukey-adjusted); PCoA on Bray-Curtis dissimilarities of community composition (D) for each age group and (H) in response to mode of birth (PERMANOVA); each point represents one stool sample; for each level of age group (D) and mode of birth (H), points are distinguished by different shapes, and ellipses indicate a 95% CI. Significance levels for each variable: ****P* < 0.001, ns: *P* > 0.05.

Supplementary Tables

Table S1. Summary of metadata and reads filtered during processing and denoising for shotgun metagenomic (A) and 18S amplicon sequencing (B). NA indicates either no samples were available or samples were removed after filtering and denoising.

Table S2. Levels of different variables under study and the number of samples per infant and per variable level after data cleaning and denoising. Due to missing data for infant P07, specifically household size and samples from 13 to 18 months, this data was excluded from the statistical analyses.

Table S3. Effects of different variables on gut microbial alpha diversity (Shannon index) in infants from a longitudinal cohort in a rural area of Morelos, Mexico. The means and standard deviations (mean ± SD) for each group are determined and compared (LMM, Wald test, Tukey-

adjusted). The marginal and conditional R^2 values, and AIC are presented for both the full model (including all variables) and the selected fitted model. Results are averaged over the posterior distributions of the fitted parameters. (-) indicates that the variable was not included in the selected model. Significance levels for each variable: ****$P < 0.0001$, ***$P < 0.001$.

Table S4. Microbial community composition homogeneity among gut samples of infants from a longitudinal cohort in a rural area of Morelos, Mexico. The average distance from the group centroid is used to quantify the multivariate dispersion of each group, and the mean distance and standard deviation (mean ± SD) are reported for each group. The significance of group differences is computed and compared (LMM, Wald test, Tukey-adjusted). The marginal and conditional R^2 values, and AIC are presented for both the full model (including all variables) and the selected fitted model. Results are averaged over the posterior distributions of the fitted parameters. (-) indicates that the variable was not included in the selected model. Significance levels for each variable: *$P < 0.05$.

Table S5. Significantly more abundant bacterial taxa (A), eukaryotic taxa (B), and bacterial pathways (C) across all age groups in infant gut samples, along with their mean relative abundance, from a longitudinal cohort in a rural area of Morelos, Mexico.
(MaAsLin2, BH-adjusted *P*). Significance levels for each variable: ****$P < 0.0001$, ***$P < 0.001$, **$P < 0.01$, *$P < 0.05$.

Table S6. Significant correlations between gut microbial variation and infant age across all age groups (A) and within each age group (B) in a longitudinal cohort of infants from a rural area of Morelos, Mexico (envfit analysis of correlations between PCoA axes and variables). Significance levels for each variable: **$P < 0.01$, *$P < 0.05$.

Table S7. Positive and negative associations among microbial taxa in age group (A) and birth mode (B) inter-kingdom co-occurrence networks. Only associations with absolute correlations > 0.4 and BH-adjusted P < 0.001 are represented, as determined by the SparCC algorithm. Bacterial phyla and eukaryotic supergroups are shaded in grey and orange, respectively. Bolded taxa indicate network hubs.

Table S8. Comparison of global and node network properties of inter-kingdom microbial co-occurrence networks across age groups (A) and modes of birth (B). The quantile used to define the most central nodes (top nodes) for each centrality measure was set to 0.75, and these node sets were then compared between networks using the Jaccard index. Metrics with significant differences are shown in bold. Significance levels for each variable: **$P < 0.01$, *$P < 0.05$.
